# Antisecretory Action of the Extract of the Aerial Parts of *Eremomastax speciosa (Acanthaceae)* Occurs through Antihistaminic and Anticholinergic Pathways

**DOI:** 10.1155/2014/323470

**Published:** 2014-02-20

**Authors:** Amang André Perfusion, Paul V. Tan, Nkwengoua Ernestine, Nyasse Barthélemy

**Affiliations:** ^1^Department of Animal Biology & Physiology, Faculty of Science, P.O. Box 812, University of Yaoundé I, Yaoundé, Cameroon; ^2^Department of Biological Sciences, Faculty of Science, P.O. Box 46, University of Maroua, Maroua, Cameroon; ^3^Department of Organic Chemistry, Faculty of Science, P.O. Box 812, University of Yaoundé I, Yaoundé, Cameroon

## Abstract

*Objective.* The objective of this study was to find out the possible antiulcer mechanism of action of *Eremomastax speciosa*. *Method.* Carbachol- and histamine-induced hypersecretion, associated with the pylorus ligation technique, were used in rats. Gastric mucosal ulceration, mucus production, pH, gastric volume, and acidity were measured. *Results.* Histamine and carbachol raised gastric acidity to 86.50 and 84.80 mEq/L, respectively, in the control rats, and the extracts (200 mg/kg) reduced gastric acidity to 34.60 and 39.00 mEq/L, respectively. Intraduodenal aqueous extract (400 mg/kg) in histamine- and carbachol-treated rats produced significant (*P* < 0.001) decreases in acid secretion to 28.50 and 28.80 mEq/L, respectively, and 100 percent inhibition of gastric ulceration. Augmented histamine-induced gastric acid secretion (90.20 mEq/L) was significantly reduced to 52.60 and 27.50 mEq/L by the 200 and 400 mg/kg doses of the aqueous extract, respectively. The extract significantly reduced (*P* < 0.001) the volume of gastric secretion and significantly increased mucus production. The ulcer inhibition potential of the extract significantly dropped to 25–44% (oral extract) and to 29–37% (duodenal extract) in carbachol/indomethacin-treated rats. *Conclusion.* The aqueous extract of *E. speciosa* has both cytoprotective and antisecretory effects. The antisecretory effect may involve a mechanism common to both cholinergic and histaminergic pathways.

## 1. Introduction

The central role of gastric acid hypersecretion in the etiology of gastroduodenal ulcers, gastrooesophageal reflux disease and gastric cancer is well known. Thus, while ulcers are almost always present in patients with Zollinger-Ellison syndrome, which is characterised by excessively high gastric acid secretion, they are absent in achlorhydric patients. The gastric acid hypersecretion can be of stress or genetic origin but can as well result from the interaction between a genetic component with environmental factors [1, 2]. Outstanding findings in the understanding of peptic ulcer etiology include the discovery histamine H_2_-receptors, the H^+^K^+^-ATPase-driven parietal cell pump by the end of the 1970s, and the more recent discovery of the role of *Helicobacter pylori* in the development of duodenal ulcer by [3]. Corresponding breakthroughs in the treatment of acid-peptic disorders include the discovery of the prototypical H_2_ antagonist, cimetidine, developed by Smith, Kline; and French (now GlaxoSmithKline) in the mid-to-late 1960s [4], the development of proton pump inhibitors [5, 6], and the development of the *H. pylori* triple therapy eradication regimens [7, 8].

The FDA-approved triple therapy regimen includes a proton pump inhibitor (Omeprazole, Lansoprazole, or pantoprazole) + Clarithromycin and Metronidazole (or Amoxicillin) or a H_2_-receptor antagonist (Cimetidine or Ranitidine) + Bismuth and antibiotics (Clarithromycin and Metronidazole (or Amoxicillin)) [9–11]. Unfortunately, *H. pylori* strains resistant to the commonly prescribed antibiotics have emerged. In addition, the high cost of the triple therapy regimen, especially for patients in poor countries, and the adverse effects associated with the antibiotics and antisecretory agents have been at the root of low patient compliance, treatment failures, and recurrence of treated ulcers [12–15]. For example, the reported adverse effects in patients taking Cimetidine involve various organ systems including the gastrointestinal system (diarrhea, constipation), central nervous system (psychosis, depression, and anxiety especially in the elderly), endocrine system (gynecomastia, reversible impotence), hepatobiliary system (dose-related increase in serum transaminases, occasional liver injury), renal system (dose-related increase in plasma creatinine) and cardiovascular system (rare cases of bradycardia and tachycardia). The three well-known disease interactions with Tagamet include liver disease, hemodialysis, and renal dysfunction, while drug interactions with Tagamet include 18 major, 336 moderate, and 317 minor ones. This litany of possible side effects associated with the most prescribed antisecretory agent underlines the current need to intensify the search for antisecretory materials from local medicinal plant sources. Other workers [16] have given a detailed description of the roles of extracellular calcium, histamine and H_2_ receptors, and acetylcholine and muscarinic (M_3_) receptors in the mechanism of gastric secretion, and our previous work [17] describes many medicinal plants with proven antisecretory activities. Some of the plants have provided active extracts, fractions, and compounds with anticholinergic or antihistaminic activity. Botanical compounds with antiulcer activity include flavonoids (i.e., quercetin, naringin, silymarin, anthocyanosides, and sophoradin derivatives), saponins (i.e., from *Panax japonicus* and *Kochia scoparia*), tannins (i.e., from *Linderae umbellatae*), gums, and mucilages (i.e., gum guar and myrrh). Among herbal drugs, liquorice, aloe gel, and capsicum (chilli) have been used extensively and their clinical efficacy documented. The cytoprotective, antioxidant, anti-inflammatory, and antiulcer activities of flavonoids, alkaloids, triterpenoids, and polyphenols have been demonstrated experimentally [17–20]. Comparative phytochemical screening of two species of *Eremomastax* revealed the presence of flavonoids, alkaloids, phenols, tannins, terpenes, and saponins [21]. A comprehensive review of drugs derived from botanical sources more commonly used or extensively studied in the world for peptic ulcer has been published [22].

*Eremomastax speciosa* (Hochst.) Cufod. (*Acanthaceae*) is widespread from West Africa through Central African Republic and N Congo-Kinshasa to S Sudan and SW Ethiopia, Madagascar. The plant is widely distributed in tropical Africa and is the only species of the genus *Eremomastax* (syn.: *Paulowilhelmia* (Lindau) and *Ruellia* (S. Moore)) [23]. It is a robust, polymorphous shrub that grows to 2 m high and has a characteristic quadrangular stem and violate underside of the leaves which has earned for it the local name *Pang nyemshe*, meaning “red on one side” in the Bamileke region of Cameroon.

The plant is commonly referred to in Cameroon as “blood plant” due to its reputed use in the treatment of cases of anemia. It is also used in Cameroonian ethnomedicine for the treatment of various stomach complaints and information from tradipractitioners suggested that it possesses antiulcer effects. The antidiarrhoeal activity of *E. speciosa* leaf aqueous extract has been reported [24]. The leaf extract is used for the treatment of male infertility among the* Ifa Nkari* People of Akwa Ibom State, Nigeria (where it is known commonly as “golden seal;” “African blood tonic plant;” local name, *Edem Ididout*, *Ndana-edem*) [25]. Its widelyclaimed antianaemic activity has been experimentally demonstrated by workers [26] who also showed anti-microbial actions against pure clinical cultures of *Staphylococcus aureus*, *E. coli*, *Candida albicans*, and *Aspergillus niger*. The Douala people of Cameroon employ *E. speciosa* variously for malaria, kidney pain, scabies anaemia diabetes, and nerves pain [27]. *E. speciosa* has been cited for its local use in the treatment of female infertility in the west region of Cameroon [28], as well as for its use in the treatment of irregular menstruation by the Aguambu-Bamumbu people of the Lebialem highlands in the South West Region of Cameroon [29]. The plant has also been cited [30] for the treatment of menstrual pains, gonorrhea, appendicitis, and dry burns and as an antipoison, and to increase and purify blood in the mount Cameroon region.

In spite of the wide gastrointestinal ethnopharmacological potential of *E. speciosa*, the only literature report to date on its antiulcer activity is the preliminary study [31] which showed that the water extract significantly inhibited the formation of HCl/ethanol-inflicted gastric lesions in rats. Recently, we have demonstrated the activity of the methanol extract against various experimental ulcer models including HCl/ethanol, absolute ethanol, cold/restraint stress, and indomethacin [32]. In the present experiment we employed the pylorus ligated technique in a series of secretagogue-induced hypersecretion models in order to study the possible mechanism of action of *E. speciosa*.

## 2. Material and Methods

### 2.1. Material

#### 2.1.1. Animals

Male albino Wistar rats (180–220 g) raised in the Animal house of the Animal Physiology laboratory, Faculty of Science, University of Yaounde I, were used. They were fed a standard laboratory diet (supplied by SPC Ltd., Bafoussam, Cameroon) and given tap water *ad libitum*. The animals were deprived of food prior to experimentation but access to water was maintained. Prior authorization for the use of laboratory animals in this study was obtained from the Cameroon National Ethics Committee (Reg. no. FWA-IRB00001954). The use, handling, and care of animals were done in adherence to the European Convention (Strasbourg, 18.III.1986) for the protection of vertebrate animals used for experimental and other purposes (ETS-123), with particular attention to Part III, articles 7, 8, and 9.

### 2.2. Methods

#### 2.2.1. Preparation of the Plant Extracts and Fractions

The aerial parts of *E. speciosa* were collected in May/June 2012 in Yaounde (centre region) and identified botanically by Paul Mezili of the Cameroon National Herbarium (by comparison with existing voucher specimen N° HNC/136984). They were chopped, rapidly dried under room temperature (25°C) for one week, and transformed into powder. 3796 g of powder was macerated in 15 L of a 2 : 1 (v/v) mixture of methanol and methylene chloride, which was the highest yielding out of the five extraction solvents tested for 72 h at room temperature. After filtration through Wattman filter paper number 3 and sequential evaporation at 40°C and 65°C in a rotavapor to remove the methylene chloride and methanol, respectively, 318 g of crude extract was obtained (8.4% yield). 159 g of crude extract was fractionated with hexane (1 L), methylene chloride (0.5 L), ethyl acetate (0.25 L), and methanol (0.125 L), respectively, to obtain 39.3 g of hexane fraction (24.7% yield), 30.5 g of methylene chloride fraction (19.2% yield), 5.6 g of ethyl acetate fraction (3.52% yield), and 3.3 g of methanol fraction (2.1% yield).

*Aqueous Extract*. 500 g of ground powder was extracted by infusion in 4 litres of boiled water for 15 minutes. After filtration through Wattman filter paper number 3, the filtrate was evaporated using a *Raven* convection air oven (Jencons-PLS, UK). The brownish solid obtained (77.7 g (15.5% yield)) was stored at 4°C. The crude extracts and fractions dissolved readily in distilled water which was used as vehicle in the subsequent experiments.

#### 2.2.2. Phytochemical Tests

Phytochemical tests for major secondary metabolites of the extracts and fractions were performed. They were screened for the presence of biologically active compounds such as alkaloids, anthocyanins, and cardiac glycosides [33]; phenols and flavonoids [34]; triterpenes and sterols [35]; saponins [36]; anthraquinones, hydrolysable, and condensed tannins [37]; and coumarins [38]. Based on the intensity of coloration or the precipitate formed during the test, secondary metabolites proportion was characterized as strongly present (+++), present (++), weakly present (+), and absent (−) when the test result was negative; it was characterized as strongly present (+++), present (++), weakly present (+) and absent (−) when the test result was negative.

#### 2.2.3. Pylorus Ligated Gastric Secretion: Screening of Extracts and Fractions for Antisecretory Activity

Fifty male rats (5 per group) were used to screen the aqueous, methanol, and methanol/methylene chloride (Me-CH_2_Cl_2_) extracts and the methylene chloride (CH_2_Cl_2_), ethyl acetate, and hexane fractions for their antisecretory potential. Following a 48 h fast, the extracts, fractions, and vehicle were administered by oral route to the test and control rats, respectively, before the experiment. One hour later, laparotomy was performed under light ether anaesthesia and the pylorus of each rat was tied and the abdominal incisions were closed. The rats were sacrificed 6 h later and the gastric juice produced by each was collected, centrifuged, and the volume measured. The ulcers formed in the glandular region of the stomachs were scored as previously described [39].

#### 2.2.4. Secretagogue-Induced Gastric Hypersecretion

Following the screening experiment, the aqueous extract, CH_2_Cl_2_ fraction, and MeOH-CH_2_Cl_2_ extract, which induced the highly significant reductions in gastric acidity, were retained for the next set of experiments. Thus, the two extracts and CH_2_Cl_2_ fraction (200 mg/kg) were administered by oral route and tested on gastric secretion induced in pylorus ligated rats by histamine (2.5 mg/kg, s.c.) or carbachol (1 mg/kg, s.c.) injected 1 h after pylorus ligature [39]. The animals were sacrificed 4 h after secretagogue administration and gastric juice was collected and ulcer indices measured. The hypersecretory effects of the secretagogues were also challenged by the aqueous extract alone (200–400 mg/kg) administered by intraduodenal route. After laparotomy and pylorus ligation, the extract was introduced into the duodenal lumen using a syringe. The stomach incisions were closed and histamine (2.5 mg/kg) or carbachol (1 mg/kg) were administered 1 h later by subcutaneous route [40]. The animals were sacrificed 4 h after secretagogue administration. Gastric juice was collected and mucus production and ulcer indices were measured.

#### 2.2.5. Augmented Histamine-Induced Gastric Secretion

A modification of the classic augmented histamine test was used to study the effect of the aqueous extract of *E. speciosa* (200 and 400 mg/kg) in rats challenged with prolonged maximal circulating levels of histamine. The test and control rats were given the extract and vehicle, respectively, by oral route 30 minutes before pylorus ligation. The positive control rats received ranitidine (100 mg/kg). Histamine dihydrochloride (1 mg/kg, s.c) was administered 4 times at hourly intervals. The gastric juice was collected 1 h after the last histamine injection and prepared for gastric acid analysis.

#### 2.2.6. Nonsteroidal Anti-Inflammatory Drug (NSAID)-Induced Gastric Ulcers in Carbachol-Treated Rats

A modification of the method of Rainsford [41] was used. Gastric ulcers were induced, following a 24 h fast, using indomethacin (30 mg/kg, s.c.) and carbachol (0.5 mg/kg, s.c.) in pylorus ligated rats. The aqueous extract of *E. speciosa* (200–400 mg/kg), ranitidine (100 mg/kg), and vehicle were administered orally 30 minutes before pylorus ligation. An hour after pylorus ligation, indomethacin (30 mg/kg, s.c.) and carbachol (0.5 mg/kg, s.c.) were administered. After 4 h the animals were sacrificed by deep ether anesthesia and the gastric secretion was collected and gastric ulceration and mucus production measured. In a second experiment, the effect of the extract administered by duodenal route was tested: immediately following pylorus ligation, the extract (200–400 mg/kg) was injected into the duodenal lumen, the abdomen stitched up, and indomethacin (30 mg/kg, s.c.) and carbachol (0.5 mg/kg, s.c.) were injected an hour later. Another 4 h later, the animals were sacrificed and the gastric secretion, gastric ulceration, and mucus production were measured.

#### 2.2.7. Measurement of Gastric Acidity

Samples of centrifuged gastric juice (1 mL) were analysed for hydrogen ion concentration by pH-metric titration with 0.1 N NaOH solution using a digital pH meter. The acid content was expressed as mEq/L.

#### 2.2.8. Mucus Production Assessment

The mucus covering of each stomach was gently scraped using a glass slide and the mucus weighed carefully using a sensitive digital electronic balance. The same experimenter performed this exercise each time.

#### 2.2.9. Statistical Analysis

Results were analysed using the one-way ANOVA followed by the Student-Newman-Keuls posttest for comparison of treatment means. *P* < 0.05 was considered significant. Values in tables are given as arithmetic means ± standard error of the mean (S.E.M).

## 3. Results

Table 1 shows the phytochemical analysis of the aqueous and MeOH/CH_2_CL_2_ extracts, hexane, methylene chloride, ethyl acetate, and methanol fractions of *E. speciosa*. The major classes of compounds present included alkaloids, flavonoids, triterpenoids, glycosides, anthocyanins, quinones, and proteins which were distributed in the different extracts and fractions.

Table 2 shows the results obtained after screening the crude extracts and the various fractions for antisecretory and cytoprotective effects. At the dose of 100 mg/kg, only the crude MeOH-CH_2_Cl_2_ and the CH_2_Cl_2_ fractions significantly reduced gastric acidity. When tested at the higher dose of 200 mg/kg, they showed significant dose-dependent reductions in gastric acidity (51 and 45% reduction, resp.) compared with the aqueous extract (49% reduction) at the same dose. Cimetidine (50 mg/kg) reduced gastric acidity by 58 percent. The low acid concentrations were accompanied by highly significant reductions (*P* < 0.001) in the volumes of gastric secretions, and significant cytoprotection was evident from the highly significant increases in mucus production compared with the controls. The MeOH-CH_2_Cl_2_ extract, the CH_2_Cl_2_ fraction, and the aqueous extract were, thus, retained for the next set of experiments.

Tables 3 and 4 show the results obtained when, in addition to pylorus ligation, gastric hypersecretion was provoked by the administration of the secretagogues, histamine, and carbachol, respectively. Administration of histamine to the control animals raised gastric acidity to 86.50 ± 2.98 mEq/L (Table 3) while carbachol-induced secretion was 84.80 ± 3.57 mEq/L (Table 4). Treatment with the extracts (200 mg/kg) and cimetidine (100 mg/kg) resulted in highly significant reductions in gastric acidity ranging from 34.60 ± 3.33 to 59.00 ± 4.08 mEq/L in the histamine-treated rats (Table 3) and from 39.00 ± 5.34 to 59.80 ± 4.49 mEq/L in the carbachol-treated rats (Table 4). The extract-induced reductions in acid secretion were accompanied by highly significant reductions in volumes of gastric juice and ulcer indices and increases in mucus production both in the histamine- and carbachol-treated rats. Due to the similarity of the results between the extracts and the high presence of chlorophyll and the residual methanol in the MeOH-CH_2_Cl_2_ extract and CH_2_Cl_2_ fraction, the aqueous extract was retained for further experimentation.

Intraduodenal administration of *E. speciosa* aqueous extract (200 and 400 mg/kg) to pylorus ligated rats subjected to histamine-induced hypersecretion produced a highly significant (*P* < 0.001) dose-related decrease of acid secretion to 28.50 ± 1.30 mEq/L and volume of gastric juice to 2.54 ± 0.13 mL for the 400 mg/kg dose. pH values increased up to 4.83 ± 0.11 for the same dose. The high dose of aqueous extract also produced 100 percent inhibition of gastric ulceration and increased mucus production to 101.69 ± 3.89 mg compared with 56.36 ± 3.35 mg for the controls (Table 5). When the aqueous extract (200 and 400 mg/kg) was administered intraduodenally to pylorus ligated rats treated with carbachol, similar dose-dependent reduction in gastric acid production to 28.80 ± 1.30 mEq/L was obtained for the 400 mg/kg dose. Gastric juice pH increased to 5.26 ± 0.12 and volume of gastric juice reduced to 2.37 ± 0.15 for the same dose. Ulcer formation was totally inhibited and gastric mucus production increased significantly for the dose of 400 mg/kg (Table 6).

Repeated histamine injection to the control rats (augmented histamine test) further raised the basal gastric acid levels to 90.20 ± 2.71 mEq/L. Pretreatment with a single oral dose of aqueous extract (200 and 400 mg/kg) prevented the histamine effect by reducing the acid secretion to values significantly below basal levels (52.60 ± 2.96 and 27.50 ± 1.29 mEq/L, resp.). pH and volume of gastric juice at the dose of 400 mg/kg were 5.02 ± 0.17 and 2.06 + 0.18 mL compared to 2.29 ± 0.06 and 7.42 ± 0.26 mL, respectively, for the controls. There was complete inhibition of ulcer formation associated with significant increase in mucus production for cimetidine (100 mg/kg) and extract (400 mg/kg) (Table 7).

The effects of the extract when administered to carbachol/indomethacin-treated rats are shown in Table 8. Coadministration of carbachol and indomethacin significantly reduced the ulcer inhibition capacity of the oral aqueous extract (200–400 mg/kg) to 25–44 % compared with 70–100 % inhibition (for carbachol alone, Table 4). This was accompanied by a drop in mucus production both for the controls and extract-treated rats. In addition, the volume of gastric juice (3.96–5.09 mL) and gastric acidity (59.5 ± 4.36–44.00 ± 4.08 mEq/L) remained high in response to the extract. When the extract was administered by duodenal route, the values of ulcer index, pH, and volumes of gastric juice remained largely unchanged, and gastric acidity reduced significantly only for the 400 mg/kg dose (Table 9).

## 4. Discussion

In a preliminary study [39] we found that the water extract of *E. speciosa* had possible cytoprotective and antisecretory effects but no study has been carried out to explore the possible mechanism of action. In the present study, several extracts and unpurified fractions were screened for comparative antisecretory effects. The MeOH-CH_2_Cl_2_ and aqueous extracts and the CH_2_Cl_2_ fraction showed significant antisecretory activity at the lower dose of 100 mg/kg and significant dose-dependent activity at 200 mg/kg and were further screened for their response to secretagogue-induced gastric acid secretion. The three products (200 mg/kg) showed highly significant reductions in acid secretion induced by histamine (31–54%) and carbachol (29–41%). However, the MeOH-CH_2_Cl_2_ extract had higher antisecretory activity against histamine-induced secretion, while the aqueous extract showed higher antisecretory effects against carbachol-induced gastric acid secretion.

The pylorus ligation technique usually causes accumulation of gastric acid in the stomach and agents that reduce acid secretion and/or increase mucus secretion are effective in inhibiting ulcer formation by this method. The accumulated acid, in addition to its corrosive action on gastric glandular epithelium, provides the optimum pH (1.6–3.2) for the conversion of pepsinogen to pepsin. Both HCl and pepsin are important ingredients for the formation of pylorus ligated ulcers [42]. The MeOH-CH_2_Cl_2_ and aqueous extracts and the CH_2_Cl_2_ fraction significantly reduced the volume and acidity of gastric secretions and increased pH of gastric juice compared with the controls. This, in addition to the significant increases in gastric mucus production, contributed to the significant inhibition of gastric ulceration (Table 2). In Shay ligated rats, gastric acid levels of 40 mEq/L and above have been associated with severe ulceration of the rat gastric mucosa [39, 43, 44]. In the present study, the extracts reduced gastric acidity from 79.4 mEq/L in the controls down to between 38.6 and 40.2 mEq/L, while inhibition of ulcer formation ranged between 74 and 100%. These results underline the protective role of the increased gastric mucus secretion in extract-treated rats (92.19–98.47 g) compared with the controls (45.04 g) (Table 1). Pepsin inactivation occurs at about pH 6. Between pH 4 and 6, pepsin is still stable but inactive [45]. Gastric pH values obtained in all the experiments in response to extract administration ranged between 3.97 and 5.26, suggesting that the extract may deactivate gastric pepsin and interfere with protein digestion.

Neural, endocrine, and paracrine systems are responsible for the physiological control of gastric secretion. Acetylcholine and histamine directly activate acid secretion but by different pathways. While acetylcholine binds to M_3_-muscarinic receptors causing an increase in parietal cell intracellular calcium, histamine binds to H_2_-receptors and provokes an elevation of both intracellular calcium and of cyclic AMP. Cimetidine, which is a well-known H_2_-receptor antagonist, inhibits the activation of adenyl cyclase, thus, blocking the formation of cyclic AMP that is necessary for HCl production. Carbachol is a cholinomimetic drug which, like acetylcholine, increases free intracellular calcium. The resulting activation of protein kinase by phosphorylation leads to increased HCl production. Degranulation of peritoneal mast cells to release histamine is critically dependent on extracellular calcium concentration. Agents like verapamil and ranitidine, that block T-type low voltage-sensitive calcium channel opening and calcium influx through interference with H^+^K^+^ ATPase from the luminal side of the stomach, reduce both the volume and acidity of gastric secretions [46–50]. Calcium channel blockers exert their inhibitory effects on histamine, gastrin, carbachol, and cyclic AMP-induced stimulation of gastric acid secretion [51].

In our study, the CH_2_Cl_2_ fraction, the MeOH-CH_2_Cl_2_ and aqueous extracts (200 mg/kg), and cimetidine significantly raised gastric pH and reduced the volume and secretion of gastric acid in both histamine- and carbachol-treated rats. Further experimentation with the aqueous extract (200–400 mg/kg) yielded highly significant dose-dependent reduction of volume and acidity of gastric secretion when the extract was administered by intraduodenal route. These results suggest that the observed reductions in gastric acidity could be due to an H_2_-receptor blocking mechanism similar to cimetidine as well as M-3 muscarinic receptor blocking activity similar to verapamil. Since carbachol- and histamine-activated acid secretions occur by separate mechanisms, the extract of *E. speciosa* may contain two or more antisecretory ingredients acting separately and synergistically. This may, therefore, explain the highly significant acid reduction observed in the secretagogue-treated rats. It is worth noting that even in histamine-treated rats, the laparotomy- and pylorus ligation-induced pain constitutes a source of stress which contributes to acid secretion through the cholinergic pathway. When histaminergic and cholinergic stimulations of gastric acid secretion were similarly reduced by the aqueous extract of *Stachytarpheta cayennensis*, it was suggested [40] that the extract could be acting through the inhibition of common steps in both pathways, possibly at the level of histamine release/H_2_-receptor interaction or at the proton pump. In addition, the effects of *E. speciosa* aqueous extract may not be attributed to a gastric luminal topical activity alone since the extract was also active when administered by intraduodenal route. The striking similarity in acid reduction capacity when the aqueous extract (200 mg/kg) was administered by oral and duodenal route both for histamine- (59.00 ± 4.08 and 54.62 ± 2.70 mEq/L, resp.) and carbachol- (50.00 ± 4.26 and 51.40 ± 3.97 mEq/L, resp.) induced secretion is suggestive of the involvement of an active secondary metabolite.

The antiulcerogenic and antisecretory effects of *E. speciosa* aqueous extract dropped when carbachol was coadministered with indomethacin. Indomethacin, an NSAID, is well-known for its ability to suppress prostaglandin synthesis, bicarbonate secretion, and gastric mucosal blood flow in animals [52–54]. These effects increase gastric mucosal susceptibility to injury and exacerbate, in synergy with carbachol, the ulcerogenic effects of increased acid, and pepsin secretion in the stomach [41, 55]. The vital role that prostaglandins play in gastric mucosal protection is well-known. When the cytoprotective effect of an antiulcer agent is significantly reduced by pretreatment with indomethacin, it is usually interpreted that the cytoprotection is mediated by endogenous prostaglandins.

In the present study, histamine-induced basal acid secretion was higher (86.5–88.3 mEq/L) compared to carbachol-induced basal secretions (84.5–85.2 mEq/L), and histamine-induced basal secretion rose to 90.2 mEq/L in the augmented histamine test. These results are in agreement with previous findings [55] which showed that carbachol-induced secretion by parietal cells is fast, small, and transient, whereas histamine-provoked secretion is slow, large, and sustained. The large sustained secretion induced by the augmented histamine test (90.20 ± 2.71 mEq/L) was reduced to very low levels by 400 mg/kg of *E. speciosa* aqueous extract (27.50 ± 1.29 mEq/L) and by 100 mg/kg of cimetidine (19.2 ± 1.68 mEq/L). Similar levels of gastric acidity (25 mEq/L) have previously been observed [39] not to cause gastric mucosal ulceration in pylorus ligated rats. Our results are similar to findings that cimetidine significantly reduces both the volume and acidity of gastric secretions. They also demonstrate the strong antisecretory and antiulcer actions of *E. speciosa* aqueous extract at 400 mg/kg similar to *Myristica fragrans* [56] and *Hibiscus rosasinensis* [7] aqueous extracts (500 mg/kg) against secretagogue-induced hyperacidity. Phytochemical screening of the extracts and fractions (Table 1) showed the predominant presence of alkaloids, triterpenes, flavonoids, and phenols which are well-known for their gastric antisecretory and cytoprotective activities [17–20].

## 5. Conclusion

In conclusion, the water and MeOH-CH_2_Cl_2_ extracts and the CH_2_Cl_2_ fraction of *E. speciosa *protected the rat gastric mucosa and inhibited gastric acid secretion. In addition to increased mucus production, the aqueous extract offers cytoprotection through a mechanism that involves the physico-chemical reenforcement of the gastric mucous layer or by effects similar to endogenous prostaglandins. The antisecretory effect of the aqueous extract may involve a mechanism common to both cholinergic and histaminergic pathways. This may be attributed to the various bioactive compounds present in the extract. The results lend credence to the wide traditional use of *E. speciosa* in the management of complaints symptomatic of peptic ulcer disease.

## Figures and Tables

**Table 1:** Preliminary phytochemical analysis of the aqueous and MeOH/CH_2_CL_2_ extracts, hexane, methylene chloride, ethyl acetate, and methanol fractions of *E. speciosa*.

	Aqueous extract	MeOH/CH_2_CL_2_ extract	Hexane fraction	CH_2_CL_2_ fraction	Ethyl acetate fraction	MeOH fraction
Tannins	+	−	−	−	−	−
Alkaloids	+++	++	−	+	−	+
Resins	++	−	−	−	−	−
Saponins	−	−	−	−	−	−
Flavonoids	+++	+++	++	++	+	+
Anthocyanins	+++	−	−	−	−	−
Phenols	+	−	−	+	−	−
Quinones	+++	++	−	++	+	−
Acids	−	+	+	+	+	−
Sugars	−	−	−	−	−	−
Oils	+	+	−	−	++	+
Coumarins	−	+	−	−	−	+
Sterols	+	++	+	++	++	−
Triterpenoids	+	+++	+	++	++	−
Glycosides	+	++	++	++	+	−
Amino acids	+	+	−	+	+	−
Proteins	+++	−	−	++	++	−

Strongly present: +++; present: ++; weakly present: +; absent: −.

**Table 2:** Effects of *E. speciosa* extracts and fractions on gastric acid secretion and ulceration in pylorus ligated rats.

Treatment	Dose (mg/kg)	Ulcer index	% Inhibition	Gastric mucus (mg)	Gastric juice (mL)	Gastric pH	Gastric acidity (mE/L)
Control	—	3.88 ± 0.21	—	45.04 ± 5.09	5.07 ± 0.20	2.66 ± 0.15	79.40 ± 5.63
CH_3_OH/CH_2_CL_2_	100	2.35 ± 0.05	39.43	78.20 ± 3.53***	2.04 ± 0.29**	3.95 ± 0.16***	46.00 ± 6.33**
CH_3_OH/CH_2_CL_2_	200	0.00 ± 0.00***	100	98.47 ± 3.67***	1.81 ± 0.13***	4.01 ± 0.17***	38.60 ± 3.90***
CH_2_CL_2_ fraction	100	3.20 ± 0.17	17.53	51.13 ± 2.95	2.14 ± 0.32***	3.54 ± 0.19**	55.60 ± 6.57*
CH_2_CL_2_ fraction	200	1.30 ± 0.37***	66.49	84.74 ± 6.06***	1.96 ± 0.10***	3.94 ± 0.09***	43.20 ± 3.65***
Eth. Acet. fraction	100	3.40 ± 0.19	12.37	60.70 ± 2.83**	5.30 ± 0.26	2.94 ± 0.06	71.60 ± 4.06
Eth. Acet. fraction	200	2.30 ± 0.38*	40.72	65.90 ± 2.33**	4.93 ± 0.36	3.40 ± 0.09	59.70 ± 3.58
Hexane fraction	100	2.77 ± 0.16*	28.60	70.72 ± 2.73***	4.54 ± 0.21	3.45 ± 0.24**	64.20 ± 6.91
Hexane fraction	200	2.40 ± 0.20	38.14	79.89 ± 3.89***	4.01 ± 0.32	3.52 ± 0.39**	54.90 ± 5.92
CH_3_OH fraction	100	3.43 ± 0.16	11.60	65.87 ± 2.70***	5.16 ± 0.21	3.06 ± 0.13	67.00 ± 6.12
CH_3_OH fraction	200	2.90 ± 0.19	25.25	67.33 ± 2.00***	5.01 ± 0.19	3.40 ± 0.25**	62.22 ± 5.76*
Aqueous extract	100	1.46 ± 0.06***	62.37	79.20 ± 3.24***	4.70 ± 0.30	3.01 ± 0.15**	63.40 ± 4.10*
Aqueous extract	200	1.00 ± 0.27***	74.23	92.19 ± 3.05***	2.10 ± 0.17***	3.97 ± 0.13***	40.02 ± 2.56***
Cimetidine	50	1.60 ± 0.43***	58.76	87.47 ± 3.76***	2.06 ± 0.28***	4.31 ± 0.15***	33.40 ± 4.32***

*N* = 5 rats per treatment; *P < 0.05, statistically significant relative to control; **P < 0.01, statistically highly significant relative to control; ***P < 0.001, statistically very highly significant relative to control.

**Table 3:** Effects of orally administered *E. speciosa* on histamine-induced gastric ulcers and gastric secretion in rats.

Treatment	Dose (mg/kg)	Ulcer index (mean + SEM)	% Inhibition	Mucus production (mg) (mean + SEM)	Volume of gastric juice (mL) (mean + SEM)	Gastric pH (mean + SEM)	Gastric acidity (mEq/L) (mean + SEM)
Control	—	4.12 ± 0.16	—	52.83 ± 2.96	6.87 ± 0.31	2.37 ± 0.04	86.50 ± 2.98
CH_3_OH/CH_2_Cl_2_	200	0.00 ± 0.00***	100.00	92.13 ± 2.89***	3.07 ± 0.18***	4.74 ± 0.34***	39.50 ± 3.19***
CH_2_Cl_2_ fraction	200	1.57 ± 0,65***	61.89	87.32 ± 6.91***	3.32 ± 0.38***	4.47 ± 0.27***	49.80 ± 4.14***
Aqueous extract	200	1.90 ± 0.29***	53.88	85.05 ± 4.21***	3.50 ± 0.45***	4.33 ± 0.23***	59.00 ± 4.08***
Cimetidine	100	0.0 ± 0.00***	100.00	88.8 ± 3.62***	2.28 ± 0.20***	4.96 ± 0.24***	34.60 ± 3.33***

*N* = 5 rats per treatment; *P < 0.05, statistically significant relative to control; **P < 0.01, statistically highly significant relative to control; ***P < 0.001, statistically very highly significant relative to control.

**Table 4:** Effects of orally administered *E. speciosa* on carbachol-induced gastric ulcers and gastric secretion in rats.

Treatment	Dose (mg/kg)	Ulcer index (mean + SEM)	% Inhibition	Mucus production (mg) (mean + SEM)	Volume of gastric juice (mL) (mean + SEM)	Gastric pH (mean + SEM)	Gastric acidity (mEq/L) (mean + SEM)
Control	—	4.03 ± 0.13	—	49.34 ± 3.20	6.89 ± 0.22	2.59 ± 0.04	84.80 ± 3.57
CH_3_OH/CH_2_Cl_2_	200	1.43 ± 0.37***	64.52	78.82 ± 3.64**	3.57 ± 0.30***	4.35 ± 0.12***	54.00 ± 4.30**
CH_2_Cl_2_ fraction	200	1.73 ± 0.50***	57.07	76.08 ± 2.83**	3.71 ± 0.25***	4.12 ± 0.14***	59.80 ± 4.49**
Aqueous extract	200	1.20 ± 0.34***	70.22	79.19 ± 3.66**	3.99 ± 0.27***	4.24 ± 0.22***	50.00 ± 4.26***
Cimetidine	50	1.10 ± 0.46***	72.71	69.42 ± 3.42*	2.76 ± 0.37***	5.48 ± 0.17***	39.00 ± 5.34***

*N* = 5 rats per treatment; *P < 0.05, statistically significant relative to control; **P < 0.01, statistically highly significant relative to control; ***P < 0.001, statistically very highly significant relative to control.

**Table 5:** Effects of duodenally administered aqueous extract of *E. speciosa* on gastric ulceration and secretion induced by histamine in rats.

Treatment	Dose (mg/kg)	Ulcer index (mean + SEM)	% Inhibition	Mucus production (mg) (mean + SEM)	Volume of gastric juice (mL) (mean + SEM)	Gastric pH (mean + SEM)	Gastric acidity (mEq/L) (mean + SEM)
Control	—	4.07 ± 0.22	—	59.06 ± 2.30	6.64 ± 0.24	2.29 ± 0.06	88.30 ± 3.56
*E. speciosa *	200	2.40 ± 0.19***	41.03	84.82 ± 2.69***	4.54 ± 0.21***	3.99 ± 0.16***	54.60 ± 2.70***
*E. speciosa *	400	0.00 ± 0.00***	100.00	101.69 ± 3.89***	2.54 ± 0.13***	4.65 ± 0.10***	28.50 ± 1.30***
Ranitidine	100	1.91 ± 0.33***	53.00	89.87 ± 2.01***	2.02 ± 0.15***	5.02 ± 0.17***	36.36 ± 1.46***

*N* = 5 rats per treatment; ***P < 0.001; statistically very highly significant relative to control.

**Table 6:** Effects of duodenally administered aqueous extract of *E. speciosa* on gastric ulceration and secretion induced by carbachol in rats.

Treatment	Dose (mg/kg)	Ulcer index (mean + SEM)	% Inhibition	Mucus production (mg) (mean + SEM)	Volume of gastric juice (mL) (mean + SEM)	Gastric pH (mean + SEM)	Gastric acidity (mEq/L) (mean + SEM)
Control	—	3.90 ± 0.19	—	56.36 ± 3.35	6.71 ± 0.19	2.72 ± 0.07	85.50 ± 2.31
*E. speciosa *	200	2.02 ± 0.20***	48.21	77.60 ± 2.78***	3.97 ± 0.25***	4.15 ± 0.12***	51.40 ± 3.97***
*E. speciosa *	400	0.00 ± 0.00***	100.00	103.20 ± 3.19***	2.37 ± 0.15***	5.26 ± 0.12***	28.80 ± 1.38***
Ranitidine	100	1.76 ± 0.23***	55.00	82.34 ± 3.48***	2.48 ± 0.19***	4.96 ± 0.28***	34.20 ± 1.42***

*N* = 5 rats per treatment; ***P < 0.001; statistically very highly significant relative to control.

**Table 7:** Effects of orally administered aqueous extract of *E. speciosa* on gastric ulceration and secretion in rats submitted to the augmented histamine test.

Treatment	Dose (mg/kg)	Ulcer index (mean + SEM)	% Inhibition	Mucus production (mg) (mean + SEM)	Volume of gastric juice (mL) (mean + SEM)	Gastric pH (mean + SEM)	Gastric acidity (mEq/L) (mean + SEM)
Control	—	4.33 ± 0.17	—	52.84 ± 2.42	7.42 ± 0.26	2.29 ± 0.06	90.20 ± 2.71
*E. speciosa *	200	2.27 ± 0.19***	47.58	82.20 ± 2.82***	3.76 ± 0.26***	3.99 ± 0.16***	52.60 ± 2.96***
*E. speciosa *	400	0.00 ± 0.0***	100.00	96.51 ± 3.12***	2.20 ± 0.27***	4.65 ± 0.10***	27.50 ± 1.29***
Ranitidine	100	0.00 ± 0.00***	100.00	73.21 ± 2.43***	2.06 ± 0.18***	5.02 ± 0.17***	19.20 ± 1.68***

*N* = 5 rats per treatment; ***P < 0.001; statistically very highly significant relative to control.

**Table 8:** Effects of orally administered aqueous extract of *E. speciosa* on gastric ulceration and secretion in pylorus-ligated rats treated with indomethacin/carbachol.

Treatment	Dose (mg/kg)	Ulcer index (mean + SEM)	% Inhibition	Mucus production (mg) (mean + SEM)	Volume of gastric juice (mL) (mean + SEM)	Gastric pH (mean + SEM)	Gastric acidity (mEq/L) (mean + SEM)
Control	—	4.13 ± 0.21	—	38.87 ± 4.59	7.32 ± 0.26	2.64 ± 0.15	92.10 ± 4.39
*E. speciosa *	200	3.10 ± 0.25**	24.94	60.63 ± 5.67*	5.14 ± 0.29***	3.96 ± 0.08***	59.50 ± 4.36***
*E. speciosa *	400	2.31 ± 0.13***	44.07	74.01 ± 6.90**	4.16 ± 0.31***	4.77 ± 0.07***	44.00 ± 4.08***
Ranitidine	100	2.03 ± 0.20***	50.85	49.02 ± 5.31	3.64 ± 0.30***	5.09 ± 0.25***	35.05 ± 3.03***

*N* = 5 rats per treatment; *P < 0.05, statistically significant relative to control; **P < 0.01, statistically highly significant relative to control; ***P < 0.001, statistically very highly significant relative to control.

**Table 9:** Effects of duodenally administered aqueous extract of *E. speciosa* on gastric ulceration and secretion in pylorus-ligated rats treated with indomethacin/carbachol.

Treatment	Dose (mg/kg)	Ulcer index (mean + SEM)	% Inhibition	Mucus production (mg) (mean + SEM)	Volume of gastric juice (mL) (mean + SEM)	Gastric pH (mean + SEM)	Gastric acidity (mEq/L) (mean + SEM)
Control	—	3.80 ± 0.13	—	44.39 ± 4.82	6.91 ± 0.15	2.72 ± 0.11	88.50 ± 5.10
*E. speciosa *	200	2.68 ± 0.23***	29.47	59.43 ± 4.45	5.02 ± 0.42***	4.02 ± 0.12***	60.50 ± 4.36***
*E. speciosa *	400	2.40 ± 0.10***	36.84	66.73 ± 5.44*	4.48 ± 0.25***	4.60 ± 0.15***	34.50 ± 3.57***
Ranitidine	100	2.03 ± 0.20***	50.85	49.02 ± 5.31	3.64 ± 0.30***	5.09 ± 0.25***	35.05 ± 3.03***

*N* = 5 rats per treatment; *P < 0.05, statistically significant relative to control; ***P < 0.001, statistically very highly significant relative to control.
